# Work-related stress and bullying: gender differences and forensic medicine issues in the diagnostic procedure

**DOI:** 10.1186/1745-6673-6-29

**Published:** 2011-11-16

**Authors:** Stefano Tonini, Andrea Lanfranco, Antonio Dellabianca, Diego Lumelli, Ines Giorgi, Fulvio Mazzacane, Camilla Fusi, Fabrizio Scafa, Stefano M Candura

**Affiliations:** 1 Department of Public Health and Neuroscience, University of Pavia, Pavia, Italy; 2 Occupational Medicine Unit, Salvatore Maugeri Foundation, Work and Rehabilitation, IRCCS, Scientific Institute of Pavia, Pavia, Italy; 3 Psychology Service, Salvatore Maugeri Foundation, Work and Rehabilitation, IRCCS, Scientific Institute of Pavia, Pavia, Italy; 4 Consultant Psychiatrist, Salvatore Maugeri Foundation, Work and Rehabilitation, IRCCS, Scientific Institute of Pavia, Pavia, Italy; 5 Department of Preclinical and Clinical Pharmacology, University of Florence, Florence, Italy

**Keywords:** psychosocial risk, mobbing, women's work, risk evaluation

## Abstract

**Background:**

The attention of international agencies and scientific community on bullying and work-related stress is increasing. This study describes the gender differences found in victims of bullying and work-related stress in an Italian case series and analyzes the critical issues in the diagnostic workup.

**Methods:**

Between 2001 and 2009 we examined 345 outpatients (148 males, 197 females; mean age: 41 ± 10.49) for suspected psychopathological work-related problems. Diagnosis of bullying was established using international criteria (ICD-10 and DSM-IV).

**Results:**

After interdisciplinary diagnostic evaluation (Occupational Medicine Unit, Psychology and Psychiatry Service), the diagnosis of bullying was formulated in 35 subjects, 12 males and 23 females (2 cases of Post-Traumatic Stress Disorder and 33 of Adjustment Disorder). Fifty-four (20 males, 34 females) suffered from work-related anxiety, while work-unrelated Adjustment Disorder and other psychiatric disorders were diagnosed in 7 and 112 subjects, respectively. Women between 34 and 45 years showed a high prevalence (65%) of "mobbing syndrome" or other work-related stress disorders.

**Conclusions:**

At work, women are more subject to harassment (for personal aspects related to emotional and relational factors) than men. The knowledge of the phenomenon is an essential requisite to contrast bullying; prevention can be carried out only through effective information and training of workers and employers, who have the legal obligation to preserve the integrity of the mental and physical status of their employees during work.

## Background

Few studies about gender differences in bullying have been carried out, despite the increasing attention of Institutions and the international scientific community. This study describes gender differences in the victims of bullying and work-related stress examined in the Occupational Medicine Unit of our Institute, and analyzes, from a forensic point of view, the critical issues still present in the diagnostic workup.

So far, the "mobbing syndrome" does not have a clear nosological definition. The ICD-10 (International Classification of Disease) and DSM-IV (Diagnostical and Statistical Manual of Mental Disorders) indicate two conditions, not necessarily work-related, that are directly related to stress: 1) the Post-Traumatic Stress Disorder (PTSD), characterized by behaviors aimed at avoiding any situation that reminds a certain problem, obsessive thinking about work issues, alertness and anxiety; and 2) the Adjustment Disorder (AD), with risk factors and clinical features similar to PTSD, but of less intensity and severity. These are also the only recognized nosological entities by the Italian workers compensation system.

Recently, attention to gender differences in employment is gradually increasing from a sectorial knowledge, concerning few interested researchers, to a widespread information [1,2].

Stress is perceived by workers as the second most important health threat and affects approximately 22% of the workforce. Also, stress-induced damage is relevant not only to individuals, but also to companies: a proportion of 50 to 60% of all working days is lost because of stress [3]. In 2002 the stress-related annual economic burden was estimated about 20 billion euro [4].

The European Parliament Resolution of 20 September 2001 identified women's work among the harassment risks. Over the years, the European Union policies have carefully taken into account the female gender factor in terms of health and safety at work. The EASHW (European Agency for Safety and Health at Work) has long encouraged the single countries to examine their issues regarding gender affecting health and safety at work, in order to plan appropriate interventions. A document of the same agency emphasizes that, compared to men, research, prevention and awareness of the risks of working women have been underestimated and neglected [1]. In the last two decades, an increasing number of women entered the working environment, but only few of them reached a leading role in decision-making. Factors such as stereotypes and discrimination, as well as bias in selection and promotion processes, can contribute to this situation.

Gender differences concerning health and safety at work are of particular interest when considering the psychosocial risk factors, especially in relation to the phenomenon of bullying [5]. A special survey of 2004, managed by the European Commission, shows that 10.2% of women and 7.3% of men have been subject to intimidation in the workplace in the previous 12 months [2]. The most affected fields are health and social services (15.7%), followed by public administration, hotels, restaurants and transport. In all considered areas of work, women suffer greater discrimination (3.1% versus 0.8% for men).

Data emerging from few studies focused on gender difference among the victims of bullying and occupational stress are somewhat conflicting [6]. A milestone was the study conducted by Vartia on prison officers, in which there was no evidence of significant gender differences in the prevalence and modes of bullying practices [7]. Some specific types of actions, as the non-allocation of work tasks and exclusion from meetings, were instead found to be more frequent against men than women. Bjorkqvist et al., reported that among victims, about 1/3 are men and 2/3 are women [8]. Also, bullying was more commonly reported by women than men (11.6 vs. 5%) in a survey conducted among businessmen [9]. This condition could result from increased exposure to negative actions, lower perceived ability to defend or to a tendency to more easily define their experience as bullying. About the perception of harassment, women mainly focus on criticisms and rumors about their private life, while men are more subjected to have their work discredited.

## Methods

From 2001 to 2009, 345 patients required a specialist visit at the Department of Occupational Medicine of our Institute for psychological health problems related, in their opinion, to bullying in the workplace. The sample consisted of 197 females (57.1%) and 148 males (42.9%), aged between 21 and 61 years (average 41 ± 10.49 years). Four subjects (1.15%) had attended the primary school only, 126 the secondary school (36.5%), 157 had a high school diploma (45.5%) and 58 had graduated (16.85%). Two hundred fifty-six patients (74.2%) were employed in private companies, while the remaining 89 (25.8%) worked at public institutions. About 13% of the subjects were executives, 15.8% intermediate managers, 45.4% clerks, 16.6% workmen, the remaining 9.5% had other qualifications.

The diagnostic process begins with an evaluation of an occupational health specialist: for each patient a careful work history is collected, as well as family, social, physiological and pathological history; this step is followed by a careful physical examination in order to identify possible diseases associated with organ disorders. Furthermore, the diagnostic protocol, developed by our Unit over the years, includes: psychological counseling, structured interview for DSM-IV: SCID (Structured Clinical Interview for DSM-IV) axis I and II, a complete personality test MMPI-2 (Minnesota Multiphasic Personality Inventory-2), psychiatric visit and other possible instrumental exams for organ disorders [10].

The structured interview is a method that, based on a specific protocol, attributes specific symptoms, on which the examiner focuses, to the different disease conditions. By giving each patient a severity score, we obtain satisfactory results. For axis I, the process starts by patient's history and leads to evaluate the presence of psychiatric disorders, such as anxiety and depression. The axis II consists of a self-report questionnaire followed by an interview regarding critical items of the questionnaire, to identify personality disorders and mental retardation.

The MMPI-2, the updated and standardized version of the MMPI test, is intended to assess the most important structural features of personality and emotional disorders. It includes 567 questions on different topics: general health, neurological conditions, cranial nerves, motility and coordination, sensitivity, vasomotor function, trophism, speech, secretory functions, cardiovascular, respiratory, gastrointestinal and genitourinary systems, habits, family and marital situation, professional activity, education, sexual, social and religious behavior, attitudes towards politics, law and order, morality, masculinity, femininity, presence of depression, manic, obsessive and compulsive disorders, presence of hallucinations, illusions, delusions, phobias, sexual sadistic and masochistic trends. The patient should respond to items with "True" or "False", but all omissions and items with dual response shall be considered as a response "I don't know." The usefulness of information obtained through the MMPI-2 depends on the ability of the subject to understand instructions, carry out the required task, understand and interpret the content of the items, and record the answers correctly. To calculate the scores, a computer program and a manual scoring are available. The interpretation of results requires a high level of psychometric, clinical, professional and characteriological competence.

The ethical committee of our Institution approved the study protocol according to the criteria of the Declaration of Helsinki.

## Results

**Figure 1:**
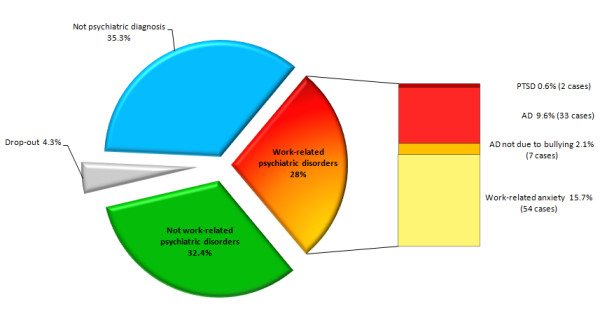
**Distribution of diagnostic conclusions in the study population**.

As shown in Figure 1, 15 (4.3%) of the 345 examined patients, did not complete the diagnostic procedures. In 122 subjects (35.3%) no psychiatric diagnosis (according to the DSM IV criteria) was formulated: among these, 104 presented altered dynamics in interpersonal relationship with colleagues and 18 concurrent stressful conditions. In 112 patients (32.4%) we diagnosed a psychiatric illness not related to work, like depressive and/or anxiety disorders, personality disorders like cluster A and B or dysthymia. Finally, 96 patients (28%) were affected by work-related psychiatric disorders: 35 (12 males, 23 females) cases of bullying including 2 cases of PTSD (2 females) and 33 of AD (12 males, 21 females) were identified and reported to the judicial and compensation authorities. Other 54 (20 males, 34 females) subjects suffered from work-related anxiety with somatization, 7 (3 males, 4 females) cases were affected by AD not consequent to bullying.

**Figure 2:**
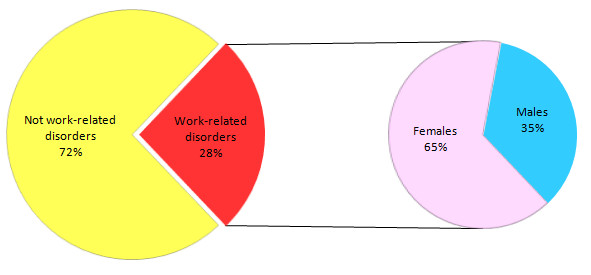
**Gender proportion in the identified cases of work-related psychiatric disorders**.

In the 96 patients with a disorder attributable to work-related stress, the most frequent diagnosis was work-related anxiety disorder (56.2% of cases), while in 34.4% was diagnosed an Adjustment Disorder and in 2.1% a Post-Traumatic Stress Disorder; thus, in 36.5% of cases was effectively diagnosed a bullying syndrome (CI = 27.5-46.4%). The average age of this subgroup was 40.6 years, with a relevant difference between men and women: 46 years for males, 39.5 for females, the latter representing a higher proportion (65%) than men (35%), which was statistically significant compared with the entire study population (p < 0.05) (Figure 2). The majority of the subjects had medium or high education; in particular, 14 patients had graduated (15.8%), 41 had a high school diploma (46.3%), the remaining 34 attended the secondary school (37.9%), nobody the primary school only. Regarding work task, 75 subjects (84.2%) worked in private companies, the remaining 14 (15.8%) in public administrations; tasks and skills were very different with a clear preponderance of office workers, in which interpersonal relationships and communication are inherently part of the work. The harassment's length was variable, ranging from 6 months to 15 years. Sixty-three subjects (70.7%) took psychoactive drugs before referral to our Department on family doctor or specialist's prescription; a person was addicted to alcohol. Finally, 3 patients were found to be civil invalids.

## Discussion and conclusions

The Acute Stress Disorder (ASD) was not considered in our study because it represents an early form of PTSD; the ASD develops within 4 weeks of the triggering event, according to the DSM-IV [11]. Moreover the Italian Law on bullying has adopted the diagnostic criteria proposed by Leymann: the diagnosis requires 6 months of harassments or 3 months of daily "attacks".

At the end of the diagnostic procedure, bullying (PTSD or AD) was actually identified in only approximately 10% of all patients, a proportion lower than that described in other case series [12]. This discrepancy could be due to different methods in the diagnostic approach or to pre-selection criteria of patients entering the outpatient service. In our series the subjects were referred by the family doctor, while in another study, the patients had been subject to a previous selection by a group of psychologists: in these subjects, the percentage of bullying diagnoses was 49% [13]. In any case, a cautionary approach should be adopted in labeling as "mobbing syndrome" clinical conditions that can show similar manifestations. In this regard, the high proportion of psychiatric diseases unrelated to work (approximately one third) must be highlighted. As recently reported, these conditions can easily generate litigation with employers, based on unfounded allegations, if superficially assessed [14]. These disputes may lead to worsening of preexisting clinical conditions. It should also be noted that even in the 35 cases identified as bullying, the diagnosis was probable and largely based on what was reported by patients; as physicians, we are not able to directly verify the existence of harassment behaviors in the workplace and the Law doesn't allow us to do that. Therefore, this is indeed a very difficult task, for which suitable methods are not yet available. Despite this limitation, our data confirm that a scientific approach to the diagnostic classification (necessarily interdisciplinary) of bullying, is crucial to correctly estimate the true prevalence of this phenomenon and to allow a proper identification from a forensic and insurance point of view.

Our results indicate that among workers with bullying there was a marked preponderance of females (65%). The highest percentage of harassed women was in the age of 34 to 45: this can be explained by the increased family commitment in this age range, resulting in rise of stressful conditions and working difficulties. The bullying behavior against a woman begins, in most cases, when she has just returned from maternity and/or needs to frequently leave work to take care of her family. In such cases, it happens that, after causing in her a deep sense of guilt for the (real or alleged) inconvenience related to her absence, the worker is isolated [15,16]. The hostility to female workers due to the use of special contractual benefits, such as particular schedules, maternity and expectancies, triggers the bullying phenomenon. Moreover, women more easily report work problems, unlike men who, according to old stereotypes, manage family through their work, thus achieving a full satisfaction. Probably because of these reasons, men are more reluctant to disclose problems related to working environment.

Females are more often affected by psychological violence and working distress such as bullying. The reasons why women are more targeted by psychological harassment may be various, e.g. a more passive attitude and rare managerial positions. In fact, bullying is mainly exerted by superiors against subordinates [17]. Finally, considering that the majority of harassed women are graduated or have a high school diploma, one can assume that the people with higher level of education are more aware or have a lower alert threshold to negative situations.

The considerable proportion of patients (33%) in which the clinical condition was likely due to work-related stress conditions, but not actually due to bullying, paves the way for some forensic considerations concerning the critical points of the diagnostic procedure in cases of suspected psychiatric disorders caused by work. A crucial point is the difficulty to obtain objective evidence of what is reported by the patient; surveys, interview with the employer, testimonies of colleagues, court proceedings, when present, are key points for the forensic evaluation of causation, in matters of bullying. Of course, a diagnosis cannot be only based on alleged bullying resulting from patient's history. Relevant information from other sources in the working environment are needed to confirm actual oppressive behaviors and harassment. Some used questionnaires do not help the medical examiner: they are often *ad hoc *self-assessment tests, in which the patient's subjective point of view stands out. Though, in clinical practice, phenomena of poor work adaptation are frequently observed, they are not always attributable to the environment, but strongly depend on the personality of the alleged victim, if not on real psycho-pathological conditions, usually belonging to persecutory or anxious-depressive disorders. These considerations strengthen the need of a thorough environmental analysis: its results are decisive for forensic determinations and must be available to make an expert judgment; data must be collected from different sources, and carefully compared, in order to evaluate the real causal relationship. The existence of bullying behavior must be thoroughly examined through analysis of reports by the different involved subjects. Information and environment description by the occupational physician is quite useful. To find credit in the law court, the oppressive behaviors must be supported by evidence, not merely suggestive or just circumstantial: when evidence is lacking, the expert must expressly ask the judge for an environmental and witness investigation. The out of court acquisition of these elements should be committed to the occupational doctor and/or to the inspection staff of the insurance agency. The expert in charge, after the suit has come to court, has often to deal with reticence of the interviewed persons, who fear being judged as taking side with or against one of the parties to the case. After documenting the type and duration of oppressive behaviors, the expert must establish the existence of a causal relationship between them and the psychic reaction of the worker, considering the pre-morbid personality of the subject and the juridical principle "*id quod plerumque accidit*".

The different types of human reactions in addressing problems or changes in work organization, as modifications in rhythms, shifts and workloads, related to productivity requirements must also be considered. These circumstances, though very common, can be interpreted and experienced by some subjects as bullying, especially in association with an increase in stress, anxiety and psychological pressure [18,19]. In other subjects, the new state of work-related stress may encourage to show new, and at times unexpected, skills; when this happens, self-esteem and confidence increases and original solutions are found to deal with difficulties. Sometimes, having one's work approved and verbally commended allows to tolerate conditions that are close to bullying, in insecure and confirmation needing individuals, who have great expectations from work and use it as vent for frustrations in other aspects of their lives.

Prevention can reduce the bullying phenomenon: the collaboration of health professionals, managers and workers representatives is needed to ensure a successful preventive action. Ethical behavior should primarily be promoted, to spread trust, tolerance and respect in the workplace [20]. Preventive action is based on the possibility of starting a cultural change in interpersonal relationships, values and attitudes. According to Ege, there are two key points:-focusing on the company, with a targeted training that adequately addresses the function of the Personnel Management Office;-furthermore, the so called "culture of litigation" (i.e. reappraising a conflict to a simple difference of opinion) should be created; individual prevention should include a personal training in dealing with conflicts by means verbal of self-defense and behavioral techniques [18].

Though these strategies may be difficult to carry out in the working environment in the whole European Union, a prevention culture, mainly promoted by occupational doctors, may be an option to limit the diffusion of bullying.

## Competing interests

The authors declare that they have no competing interests.

## Authors' contributions

ST conceived of the study, participated in its design and coordination, edited and reviewed the manuscript. AL conceived of the study, participated in its design and coordination, prepared the figures, edited and reviewed the manuscript. AD analyzed the collected data, participated in the linguistic revision. DL participated in the design of the study, analyzed and interpreted the collected data, performed the statistical analysis and prepared the figures. IG collected, analyzed and interpreted the data. FM collected, analyzed and interpreted the data. CF collected, analyzed and interpreted the data, performed the statistical analysis. FS participated in the design of the study and its coordination. SMC conceived of the study, participated in its design and coordination, revised the final document. All authors read and approved the final manuscript.
